# Supplementary material for: Explainable Artificial Intelligence in Neuroimaging of Alzheimer’s Disease
Source: Diagnostics (Basel). 2025 Mar 4;15(5):612. doi: 10.3390/diagnostics15050612 (PMC11899653; doi:10.3390/diagnostics15050612)
Supplement: Supplementary file 1 [file diagnostics-15-00612-s001.zip › diagnostics-3403159-supplementary.pdf]

Table S1. Search syntax.

Database	Search strategy	Results
PubMed	((XAI) OR (Explainable Artificial Intelligence) OR (Explainable Machine Learning) OR (Interpretable Machine Learning)) AND ((Alzheimer Disease [MeSH]) OR (Senile Dementia) OR (Alzheimer Dementia) OR (Alzheimer Sclerosis) OR (Alzheimer Syndrome) OR (Alzheimer's Diseases) OR (AD) OR (Alzheimer-Type Dementia) OR (Presenile Dementia) OR (Primary Senile Degenerative Dementia))	**256**
IEEE Xplore	((("All Metadata":"XAI") OR ("All Metadata":"Explainable Artificial Intelligence") OR ("All Metadata":"Explainable Machine Learning") OR ("All Metadata":"Interpretable Machine Learning")) AND (("All Metadata":"Alzheimer Dementia") OR ("All Metadata":"Alzheimer Sclerosis") OR ("All Metadata":"Alzheimer Syndrome") OR ("All Metadata":"Alzheimer Type Senile Dementia") OR ("All Metadata":"Alzheimer's Diseases") OR ("All Metadata":"Alzheimer-Type Dementia") OR ("All Metadata":"AD") OR ("All Metadata":"Presenile Dementia") OR ("All Metadata":"Primary Senile Degenerative Dementia") OR ("All Metadata":"Senile Dementia") OR ("All Metadata":"Alzheimer")))	**82**

Table S2. Data extraction table of the articles obtained from the search results.

**Author Year**	**XAI Task**	**XAI Algorithm**	**AD Dataset**	**Result Parameter and Outcome**
Amoroso et al.[1] 2023	Structural brain connectivity analysis and classification in AD and MCI patients using ML models	SHAP	ADNI	- RF achieved the highest model performance, with a mean three-class accuracy of 0.66 ± 0.07.<br>- Explainability analyses highlight the contributions of regions like the putamen, middle temporal gyrus, and superior temporal gyrus; from a class-related perspective, it is possible to identify specific areas such as the hippocampus and amygdala for AD and the posterior cingulate and precuneus for MCI.
Leandro et al.[2] 2023	Analyzing the MRI pixel intensity change following the texture analysis of extracted entorhinal cortex and hippocampus in AD, MCI, and control subjects	SHAP	ADNI	- XGBoost achieved f1-scores of 0.949, 0.818, and 0.810 for NC vs. AD, MC vs. MCI, and MCI vs. AD, respectively.<br>- SHAP plots show entorhinal cortex (contrast, sum average, volume) and hippocampal (variance, sum variance) features were often the highest-impact predictors. Lower ERC/hippocampal volume shifted classification toward AD; subtler volume differences appeared between NC and MCI.
Bogdanovic et al.[3] 2022	Conduct a comprehensive analysis that includes medical, cognitive, and lifestyle data to achieve both global and local interpretability of the intelligent model and draw conclusions regarding the established hypothesis	SHAP	ADNI	- XGBoost produced an f1-score of 0.84.<br>- The study's main findings reveal that the CDRSB feature significantly impacts the model's output, followed by other cognitive test results. Gender and APOE4 features have the least influence.<br>- Low CDRSB, high values of MMSE and RAVLT_immediate tend to have a significant positive impact on a CN diagnosis.
De Santi et al.[4] 2022	Performing multiclass classification on volumetric 18F-FDG PET images for the diagnosis of AD	Saliency Map (SM) and Layerwise Relevance Propagation (LRP)	ADNI	- The model attained AUC values of 0.81 for CN, 0.63 for MCI, and 0.77 for AD.<br>- Statistical inference identified significant differences in SM/LRP distributions between different classes in specific anatomical regions.<br>- The average importance metrics for brain regions were highlighted using a gradient colormap, with higher values indicating greater importance.<br>- Post hoc comparisons revealed significant differences in registered PET scans across different class combinations.
Hernandez et al.[5] 2022	Investigating the models of the top three TADPOLE Challenge methods (XGB, RF, and SVM) within a unified framework	SHAP	ADNI	- Diagnosis was found to be the most significant feature for all three methods.<br>- XGB is recommended to be used with model ensembles due to the variability of the XGB-selected features.

				- RF models mainly rely on cognitive information.<br>- The importance given to different features for SVM strongly changes among different bootstraps, which questions the suitability of SVM for the computer-aided diagnosis problem.
Jahan et al.[6] 2023	Enhancing the accuracy of AD diagnosis by integrating clinical data, MRI segmentation data, and psychological assessments	SHAP	OASIS	- RF had a 10-fold cross-validation accuracy of 98.81% for predicting five classes of AD.<br>- For Memory, mean (SHAP) is about 12.33% on AD (Class 1), 42.47% on CN (Class 0), 21.92% on Uncertain (Class 3), 6.85% on Non-AD (Class 2), and 16.44% on Others (Class 4), meaning Memory has more influence on predicting CN rather than the rest of the classes.
Mahmud et al.[7] 2024	Proposing XAI-based methodology for diagnosing AD, also using deep transfer learning and ensemble modeling	Saliency Maps and Grad-CAM	Kaggle	- Achieving an accuracy of 96%, incorporating explainable AI techniques.<br>- Providing visual insights into the neural regions influencing the diagnosis.
Zhang et al.[8] 2021	Diagnosis of AD using an explainable 3D Residual Self-Attention Deep Neural Network	Grad-CAM	ADNI	- The model achieved an accuracy of 0.913+0.012.<br>- The study used 3D Grad-CAM to visually identify and highlight the important areas in the brain that contribute to the model's decisions.
Sarica et al.[9] 2024	Gender-related variations in forecasting the advancement from MCI to AD	SHAP	ADNI	- Global comparisons between male and female models showed limited similarity (below 71.1%).<br>- FDG and HCI emerged as the most common features both male and female models share.<br>- The average predicted risk calculated by SHAP for male MCI patients (E[f(x)] = 1.769) was lower than that for female MCI patients 2.534.
Song et al.[10] 2024	Investigate the specific region of interest (ROI) in the brain that the model highlights regarding differences in these ROIs among individuals with AD and control	Grad-CAM	ADNI	- The model reached a top accuracy of 96.4% on the test set.<br>- The explainability results indicated that the regions of interest (ROIs) were mainly found in the precuneus and hippocampus for AD subjects, whereas the models analyzed the entire brain for the control group.
Vetrithangam et al.[11] 2024	Create a new and interpretable deep learning model for quick and precise AD identification	Grad-CAM, SHAP, and LIME	ADNI and OASIS	- The model achieved an accuracy of 99.39%, a recall of 99.47%, and a specificity of 99.3% on the ADNI dataset. Additionally, it reached an accuracy of 99.36%, a recall of 99.53%, and a specificity of 99.15% on the OASIS dataset.
Coluzzi et al.[12] 2025	Measuring the effectiveness of models against established AD biomarkers quantitatively	Grad-CAM	OASIS	- ResNet18 revealed notable differences between AD and control across 70 brain regions. In contrast, BC-GCN-SE identified significant differences between AD and control in 46 parcels.<br>- Twenty-two parcels displayed concordant significance between both methods, predominantly in cortical areas, with two exceptions in the cerebellum.
García-Gutiérrez et al.[13] 2024	Explore the potential for modeling additional dimensions of disease data to identify patients with MCI who are likely to progress to dementia, including variations in brain metabolism evaluated through FDG-PET	Integrated Gradients (IG)	ADNI	- Findings indicate a significant association between hypometabolism, progression of disease, and cognitive decline.<br>- Using XAI techniques emphasized the crucial roles of the hippocampus, cingulate cortex, and subcortical structures in the model predictions.
Cai et al.[14] 2023	Enhance the prediction of MCI-to-AD progression by utilizing XAI models that ensure interpretability	Explainable Boosting Machine (EBM)	ADNI	- The five-year prediction accuracy was 85% (AUC = 0.92).<br>- MRI markers are crucial in the initial stages of AD, whereas cognitive scores are more significant during the middle stages.<br>- The EBM framework offers insights at both global and local levels of interpretability.
Brusini et al.[15] 2024	Investigate the dMRI Apparent Measures Using Reduced Acquisitions (AMURA) as imaging indicators for brain microstructural changes that happen in the initial phases of AD	SHAP and LIME	ADNI	- The best SVM classification achieved an accuracy of 0.73.<br>- The explainability analysis indicated stability in the results and highlighted the cingulum's key role in signaling early signs of progressive changes in AD.

				- The left cingulum linking the hippocampus was identified as the key white matter (WM) tract that differentiates individuals with amyloid/tau positivity from those who are negative.
Saad Saoud et al.[16] 2024	Utilizing MRI data to predict AD and MCI through a framework that incorporates region-of-interest methodology and deep learning	3D Vision Transformers and Region of Interest	ADNI	- The model improves explainability by dividing MRI brain scans into 138 unique volumes corresponding to relevant clinical subregions.<br>- This model not only reaches top performance in AD classification but also markedly enhances explainability and interpretability, thus becoming a crucial tool for clinicians.
AbdelAziz et al.[17] 2024	Present the Squeeze-and-Excitation Convolutional Neural Network combined with a random forest framework for the early detection of AD using MRI scans	Saliency Maps	Kaggle dataset	- SECNN-RF demonstrates high accuracy (99.89%) and offers an explainable analysis, enhancing the model's interpretability.<br>- Mild dementia saliency maps highlight the hippocampus and nearby cortical areas. Moderate dementia saliency maps show more intensely highlighted locations in the hippocampus and more significant cortical regions than mild dementia maps. The saliency map for individuals without dementia and those with very mild dementia reveals minor findings in hotspots.
Bhattarai et al.[18] 2024	Present the Deep-SHAP approach to explore the multivariate connections between regional imaging metrics and cognitive function	SHAP	OASIS	- Applying MRI data, Deep-SHAP highlighted the insula, lateral occipital cortex, medial frontal cortex, temporal pole, and occipital fusiform gyrus as key contributors to global cognitive decline in MCI/AD. Similarly, using amyloid PiB-PET data, Deep-SHAP identified the inferior temporal, parahippocampal, inferior frontal, supratemporal, and lateral frontal gray matter as critical regions.
Bapat et al.[19] 2023	Predict the transition from MCI to AD within a four-year period by utilizing longitudinal whole-brain 3D MRI and neurocognitive assessments	Grad-CAM	ADNI	- The model achieved an accuracy of 0.834, a significant improvement compared to models trained on a single timepoint or modality.<br>- The MRI regions crucial for prediction included the putamen, thalamus, amygdala, frontal pole, frontal gyrus, and planum polare.
Kim [20] 2023	Utilizing graph convolutional networks (GCNs) in a correlation-based population graph to improve AD prognosis and reveal its progression complexities	GNNExplainer	ADNI	- Correlation-based GCN (GCN-corr) yielded the highest AUCs vs. random-edge GCN and conventional ML (SVM/RF/LR/MLP).<br>- GNNExplainer identified key features per subgroup (e.g., left precentral gyrus, precuneus, APOE ε4, age, education).
De Francesco et al.[21] 2023	Develop and interpret a machine learning algorithm that can distinguish between AD, dementia with Lewy bodies, frontotemporal dementia, and cognitively healthy individuals	SHAP	Combined data from ADNI, FTLDNI, NACC, PDBP, Newcastle	- Overall: accuracy = 87.5%, precision = 88.0%, recall = 88.36%, F1 = 87.88%, AUC = 97.79%.<br>- SHAP analyses revealed the highest-impact features: CDR, corticospinal tract FA, hippocampal volume, entorhinal thickness, fronto-occipital fasciculus, etc.
Khan et al.[22] 2024	Explainable multiclass classification of AD severity and using genetic programming (GP) technique for reducing Rician noise	SHAP	Kaggle MRI dataset	- Final classification into four classes achieved up to 96.4% accuracy.<br>- SHAP maps show which image regions are most critical for distinguishing each severity level.
Khan et al.[23] 2024	Generalized fractional-order CNN classifier with XAI capabilities is proposed for accurate and interpretable classification of AD	LIME	ADNI	- The Fr-CNN model attained an accuracy of 99%, surpassing other models.<br>- LIME enhances model transparency, which boosts the model's analytic capabilities and proficiencies.
Adarsh et al.[24] 2024	Diagnostic framework that combines CNNs with Multi-feature Kernel Supervised Discriminative Dictionary Learning (MKSCDDL), allowing accurate classification of AD, MCI, and CN individuals	LIME and CAM	ADNI	- The Explainable CNN + MKSCDDL model achieves an accuracy of 98.27%, an AUC of 0.982, and sensitivity and specificity values of 98.87% and 96.46%, respectively.<br>- LIME and CAM integration offers transparency and interpretability, which are often missing in deep learning, assisting healthcare practitioners in identifying brain regions impacted by cognitive disorders.

1. Amoroso, N.; Quarto, S.; La Rocca, M.; Tangaro, S.; Monaco, A.; Bellotti, R. An explainability artificial intelligence approach to brain connectivity in Alzheimer's disease. *Frontiers in Aging Neuroscience* **2023**, *15*, 1238065.
2. Leandrou, S.; Lamnisos, D.; Bougias, H.; Stogiannos, N.; Georgiadou, E.; Achilleos, K.; Pattichis, C.S.; Initiative, A.s.D.N. A cross-sectional study of explainable machine learning in Alzheimer’s disease: diagnostic classification using MR radiomic features. *Frontiers in Aging Neuroscience* **2023**, *15*, 1149871.
3. Bogdanovic, B.; Eftimov, T.; Simjanoska, M. In-depth insights into Alzheimer’s disease by using explainable machine learning approach. *Scientific Reports* **2022**, *12*, 6508.
4. De Santi, L.A.; Pasini, E.; Santarelli, M.F.; Genovesi, D.; Positano, V. An explainable convolutional neural network for the early diagnosis of Alzheimer’s disease from 18F-FDG PET. *Journal of Digital Imaging* **2023**, *36*, 189-203.
5. Hernandez, M.; Ramon-Julvez, U.; Ferraz, F.; Consortium, w.t.A. Explainable AI toward understanding the performance of the top three TADPOLE Challenge methods in the forecast of Alzheimer’s disease diagnosis. *PloS one* **2022**, *17*, e0264695.
6. Jahan, S.; Abu Taher, K.; Kaiser, M.S.; Mahmud, M.; Rahman, M.S.; Hosen, A.S.; Ra, I.-H. Explainable AI-based Alzheimer’s prediction and management using multimodal data. *Plos one* **2023**, *18*, e0294253.
7. Mahmud, T.; Barua, K.; Habiba, S.U.; Sharmen, N.; Hossain, M.S.; Andersson, K. An explainable AI paradigm for Alzheimer’s diagnosis using deep transfer learning. *Diagnostics* **2024**, *14*, 345.
8. Zhang, X.; Han, L.; Zhu, W.; Sun, L.; Zhang, D. An explainable 3D residual self-attention deep neural network for joint atrophy localization and Alzheimer’s disease diagnosis using structural MRI. *IEEE journal of biomedical and health informatics* **2021**, *26*, 5289-5297.
9. Sarica, A.; Pelagi, A.; Aracri, F.; Arcuri, F.; Quattrone, A.; Quattrone, A.; Initiative, A.s.D.N. Sex differences in conversion risk from mild cognitive impairment to Alzheimer’s disease: an explainable machine learning study with random survival forests and SHAP. *Brain Sciences* **2024**, *14*, 201.
10. Song, B.; Yoshida, S.; Initiative, A.s.D.N. Explainability of three-dimensional convolutional neural networks for functional magnetic resonance imaging of Alzheimer’s disease classification based on gradient-weighted class activation mapping. *Plos one* **2024**, *19*, e0303278.
11. Vetrithangam, D.; Arunadevi, B.; Pegada, N.K.; Mehta, A.; Kumar, P.; Parihar, P.; Selvakumar, S. Towards Explainable Detection of Alzheimer's Disease: A Fusion of Deep Convolutional Neural Network and Enhanced Weighted Fuzzy C-Mean. *Current medical imaging* **2024**, *20*, e15734056317205.
12. Coluzzi, D.; Bordin, V.; Rivolta, M.W.; Fortel, I.; Zhan, L.; Leow, A.; Baselli, G. Biomarker Investigation Using Multiple Brain Measures from MRI Through Explainable Artificial Intelligence in Alzheimer’s Disease Classification. *Bioengineering* **2025**, *12*, 82.
13. García-Gutiérrez, F.; Hernández-Lorenzo, L.; Cabrera-Martín, M.N.; Matias-Guiu, J.A.; Ayala, J.L.; Initiative, A.s.D.N. Predicting changes in brain metabolism and progression from mild cognitive impairment to dementia using multitask Deep Learning models and explainable AI. *NeuroImage* **2024**, *297*, 120695.
14. Cai, J.; Hu, W.; Ma, J.; Si, A.; Chen, S.; Gong, L.; Zhang, Y.; Yan, H.; Chen, F.; Initiative, A.s.D.N. Explainable Machine Learning with Pairwise Interactions for Predicting Conversion from Mild Cognitive Impairment to Alzheimer’s Disease Utilizing Multi-Modalities Data. *Brain Sciences* **2023**, *13*, 1535.

15. Brusini, L.; Cruciani, F.; Dall’Aglio, G.; Zajac, T.; Galazzo, I.B.; Zucchelli, M.; Menegaz, G. XAI-Based Assessment of the AMURA Model for Detecting Amyloid–β and Tau Microstructural Signatures in Alzheimer’s Disease. *IEEE Journal of Translational Engineering in Health and Medicine* **2024**.
16. Saoud, L.S.; AlMarzouqi, H. Explainable early detection of Alzheimer’s disease using ROIs and an ensemble of 138 3D vision transformers. *Scientific Reports* **2024**, *14*, 27756.
17. AbdelAziz, N.M.; Said, W.; AbdelHafeez, M.M.; Ali, A.H. Advanced interpretable diagnosis of Alzheimer's disease using SECNN-RF framework with explainable AI. *Frontiers in Artificial Intelligence* **2024**, *7*, 1456069.
18. Bhattarai, P.; Thakuri, D.S.; Nie, Y.; Chand, G.B. Explainable AI-based Deep-SHAP for mapping the multivariate relationships between regional neuroimaging biomarkers and cognition. *European Journal of Radiology* **2024**, *174*, 111403.
19. Bapat, R.; Ma, D.; Duong, T.Q. Predicting Four-Year’s Alzheimer’s Disease Onset Using Longitudinal Neurocognitive Tests and MRI Data Using Explainable Deep Convolutional Neural Networks. *Journal of Alzheimer's Disease* **2024**, 1-11.
20. Kim, S.Y. Personalized Explanations for Early Diagnosis of Alzheimer’s Disease Using Explainable Graph Neural Networks with Population Graphs. *Bioengineering* **2023**, *10*, 701.
21. De Francesco, S.; Crema, C.; Archetti, D.; Muscio, C.; Reid, R.I.; Nigri, A.; Bruzzone, M.G.; Tagliavini, F.; Lodi, R.; D’Angelo, E. Differential diagnosis of neurodegenerative dementias with the explainable MRI based machine learning algorithm MUQUBIA. *Scientific reports* **2023**, *13*, 17355.
22. Khan, S.U.; Albanyan, A.; Bilal, M.; Ullah, S. A genetic programming Rician noise reduction and explainable deep learning model for Alzheimer's diseases severity prediction. *Journal of Alzheimer’s Disease* **2024**, *102*, 129-142.
23. Khan, Z.A.; Waqar, M.; Chaudhary, N.I.; Raja, M.J.A.A.; Khan, S.; Khan, F.A.; Chaudhary, I.I.; Raja, M.A.Z. Fractional gradient optimized explainable convolutional neural network for Alzheimer's disease diagnosis. *Heliyon* **2024**, *10*.
24. Adarsh, V.; Gangadharan, G.; Fiore, U.; Zanetti, P. Multimodal classification of Alzheimer's disease and mild cognitive impairment using custom MKSCDDL kernel over CNN with transparent decision-making for explainable diagnosis. *Scientific Reports* **2024**, *14*, 1774.
